# Differences in adolescent activity and dietary behaviors across home, school, and other locations warrant location-specific intervention approaches

**DOI:** 10.1186/s12966-020-01027-1

**Published:** 2020-09-29

**Authors:** Adrian Ortega, Carolina M. Bejarano, Christopher C. Cushing, Vincent S. Staggs, Amy E. Papa, Chelsea Steel, Robin P. Shook, Debra K. Sullivan, Sarah C. Couch, Terry L. Conway, Brian E. Saelens, Karen Glanz, Lawrence D. Frank, Kelli L. Cain, Jacqueline Kerr, Jasper Schipperijn, James F. Sallis, Jordan A. Carlson

**Affiliations:** 1 Clinical Child Psychology Program and Schiefelbusch Institute for Life Span Studies, University of Kansas, 1000 Sunnyside Avenue, Lawrence, Kansas, USA; 2 Center for Children’s Healthy Lifestyles and Nutrition, Children’s Mercy Kansas City, 610 E. 22nd Street, Kansas City, MO USA; 3 Biostatistics & Epidemiology, Health Services & Outcomes Research, Children’s Mercy Kansas City, Kansas City, MO USA; 4 School of Medicine, University of Missouri-Kansas City, Kansas City, USA; 5 Department of Dietetics and Nutrition, University of Kansas Medical Center, Kansas City, Kansas USA; 6 Department of Rehabilitation, Exercise and Nutrition Sciences, College of Allied Health Sciences, University of Cincinnati, Cincinnati, OH USA; 7 Department of Family Medicine and Public Health, University of California San Diego, San Diego, California USA; 8 Department of Pediatrics, University of Washington & Seattle Children’s Research Institute, Seattle, Washington USA; 9 Perelman School of Medicine and School of Nursing, University of Pennsylvania, Philadelphia, PA USA; 10 School of Community and Regional Planning, University of British Columbia, Vancouver, British Columbia Canada; 11 Department of Sports Science and Clinical Biomechanics, University of Southern Denmark, Odense, Denmark; 12 Mary MacKillop Institute for Health Research, Australian Catholic University, Melbourne, Australia

**Keywords:** Built environment, Nutrition, Obesity, Physical activity, Sedentary behavior, Adolescents

## Abstract

**Background:**

Investigation of physical activity and dietary behaviors across locations can inform “setting-specific” health behavior interventions and improve understanding of contextual vulnerabilities to poor health. This study examined how physical activity, sedentary time, and dietary behaviors differed across home, school, and other locations in young adolescents.

**Methods:**

Participants were adolescents aged 12–16 years from the Baltimore-Washington, DC and the Seattle areas from a larger cross-sectional study. Participants (*n* = 472) wore an accelerometer and Global Positioning Systems (GPS) tracker (Mean days = 5.12, SD = 1.62) to collect location-based physical activity and sedentary data. Participants (*n* = 789) completed 24-h dietary recalls to assess dietary behaviors and eating locations. Spatial analyses were performed to classify daily physical activity, sedentary time patterns, and dietary behaviors by location, categorized as home, school, and “other” locations.

**Results:**

Adolescents were least physically active at home (2.5 min/hour of wear time) and school (2.9 min/hour of wear time) compared to “other” locations (5.9 min/hour of wear time). Participants spent a slightly greater proportion of wear time in sedentary time when at school (41 min/hour of wear time) than at home (39 min/hour of wear time), and time in bouts lasting ≥30 min (10 min/hour of wear time) and mean sedentary bout duration (5 min) were highest at school. About 61% of daily energy intake occurred at home, 25% at school, and 14% at “other” locations. Proportionately to energy intake, daily added sugar intake (5 g/100 kcal), fruits and vegetables (0.16 servings/100 kcal), high calorie beverages (0.09 beverages/100 kcal), whole grains (0.04 servings/100 kcal), grams of fiber (0.65 g/100 kcal), and calories of fat (33 kcal/100 kcal) and saturated fat (12 kcal/100 kcal) consumed were nutritionally least favorable at “other” locations. Daily sweet and savory snacks consumed was highest at school (0.14 snacks/100 kcal).

**Conclusions:**

Adolescents’ health behaviors differed based on the location/environment they were in. Although dietary behaviors were generally more favorable in the home and school locations, physical activity was generally low and sedentary time was higher in these locations. Health behavior interventions that address the multiple locations in which adolescents spend time and use location-specific behavior change strategies should be explored to optimize health behaviors in each location.

## Introduction

In the United States (US), one in every five adolescents is obese [1]. Fewer than 25% of US adolescents engage in the recommended amounts of physical activity [2] and the overall dietary quality of US adolescents is suboptimal [3]. Youth who develop obesity are at a greater risk for adverse health effects in adulthood such as cardiovascular disease and Type 2 diabetes [4–6]. The formation (or lack thereof) of healthy behaviors in adolescence, including physical activity, sedentary time, and dietary behaviors can carry over into adulthood. Therefore investigation of factors related to these health behaviors during adolescence is critical [7–9].

Ecological models suggest that health behaviors are influenced by individual-level and broader contextual factors including social/cultural, built environment, and policy factors [10]. As youth spend time in multiple locations each day, with large amounts of time commonly spent at home and at school, the contextual factors within these locations may differentially cue, support, or constrain physical activity and dietary behaviors. For instance, schools may encourage physical activity through physical education classes and recess time but also contribute to prolonged sedentary time and adverse dietary behaviors [11, 12]. Examination of how locations relate to health behaviors is warranted given that opportunities for engaging in active living and healthy eating can differ substantially across locations and the relative influence of location versus individual factors on health behaviors is still unclear [10]. While previous literature has demonstrated differences in where youth accumulate moderate-to-vigorous physical activity (MVPA) [13–17], supporting the importance of schools, neighborhoods, greenspaces, and active commuting locations, less is known regarding how sedentary time and dietary behaviors vary across locations.

It is conceivable that total sedentary time and *sedentary patterns*, such as time spent in prolonged sedentary bouts, may vary across locations. For example, since youth spend a majority of their school day in classrooms that promote sitting and being sedentary [18], it is likely that youth spend more time in prolonged sedentary bouts or take fewer breaks from sedentary time when at school [12]. At home, there are many opportunities for sedentary leisure, homework, or work time, but adolescents are typically more able to move around. Locations outside of the school and home may support more (e.g., restaurants, movie theaters) or less (e.g., parks, recreation areas) sedentary time. Given the mounting evidence regarding the importance of both total sedentary time and sedentary patterns in relation to health risk factors [19–21], understanding locations that support or limit these behaviors is critical for targeted disease prevention efforts.

Adolescent eating occurs in multiple locations, and availability of unhealthy versus healthy foods can differ across locations, potentially resulting in location-specific dietary behaviors [22, 23]. For example, adolescents can have access to fewer food options at school compared to when at home, where food options are typically more plentiful but potentially more regulated by parents [24, 25]. Evidence is accumulating on the nutritional impact of eating at restaurants and fast food outlets, which has been associated with higher energy intake and poorer dietary quality in adolescents [26–29]. While some studies have examined the quality of foods *provided* in schools and other single locations [29–31], fewer studies have investigated dietary *consumption* at school relative to other locations within the same individual in adolescents.

This cross-sectional study first aimed to examine whether physical activity, sedentary time, and indicators of healthy and unhealthy dietary behaviors differed across home, school, and other locations as captured objectively (physical activity and sedentary time) or from multiple 24-h recalls with reported eating location information (dietary behaviors). This aim advances previous work by simultaneously examining multiple health behaviors across locations to comprehensively understand locational patterns for these behaviors. The study’s second aim examined associations of these behaviors between each pair of locations (e.g., school versus home, school versus other, and home versus other) to identify the extent to which adolescents who engaged in healthier behaviors in one location were more likely to engage in healthier behaviors in other locations. These analyses were performed to improve understanding of the relative role of location versus individual factors in shaping health behaviors. While differences in MVPA across locations in this sample has been presented previously [13], in the present paper, locations were grouped differently so that the same locations could be investigated between physical activity and dietary analyses. These methods also help show whether location-based differences are similar for physical activity, sedentary time, and dietary behaviors or whether each health behavior has a distinct pattern across locations.

## Methods

### Participants and procedures

Participants for the present analyses were part of the Teen Environment and Neighborhood (TEAN) Study examining the relation of built environmental factors with physical activity and dietary behaviors [32]. Healthy adolescents between the ages of 12–16 years and one of their parents were recruited from the Baltimore, MD-Washington, DC and the Seattle-King County, WA metropolitan areas in 2009–2011. Participants were recruited from 447 census block groups and were evenly stratified across census block groups representing high or low neighborhood walkability and high or low median household income, resulting in four (2 × 2) study design quadrants [32]. Measurement occurred only during the school year and assessments of participants were balanced by season across study quadrants.

Households were contacted by phone, and eligible participants were invited to participate in the study. The overall participation rate was 36%, which was comparable across study quadrants. Participants were phone screened by the research staff and subsequently excluded from the study if they had any physical, medical, or cognitive impairments that would limit their physical activity, affect their dietary behaviors, or impact their ability to complete measures. Eligible participants were mailed consent and assent forms, which were followed up with a phone call by a research assistant to answer questions. After informed consent and assent forms were received, adolescent participants were asked to wear an accelerometer and Global Positioning System (GPS) tracker for 7 days during waking hours. Participants earned US $40 financial compensation for completing study procedures. The study was approved by the Institutional Review Board at San Diego State University and the University of California, San Diego.

A total of 928 adolescents participated in the TEAN study. Analyses in the present paper involving physical activity/sedentary variables excluded participants who did not receive a GPS tracker or never recorded any data (*n* = 130), whose home address was not available in the geocoding database (*n* = 29), or who did not provide their school’s name/address or were homeschooled (*n* = 93). Adolescents who did not wear both the GPS and accelerometer devices together for ≥1 valid school day and ≥ 1 valid weekend day (*n* = 204) were also excluded to obtain estimates of behaviors on both weekday (school) and weekend days. Valid days were defined as those with ≥8 h of concurrent data from both devices, and school days were defined as weekdays during which the participant spent ≥200 min at their school as measured by the GPS. Analysis involving dietary variables excluded participants who did not complete dietary recalls on ≥1 school day and ≥ 1 weekend day (*n* = 139). Therefore, the current sample comprised 472 adolescents for physical activity and sedentary variable analyses, and 789 adolescents for dietary analyses.

### Measures

#### Demographics

Adolescents self-reported demographic information including their age, sex, and race/ethnicity (dichotomized as white non-Hispanic versus non-white or Hispanic). Parents reported the highest level of education attained by any adult in the household (dichotomized as college degree versus less education), their marital status (married/living with partner versus other), and their approximate annual household income.

#### Anthropometrics

Adolescents self-reported their height and weight. Participants were asked to take the measurements at home (with instructions provided) or use doctor or school measurements taken within the last month.

#### GPS tracking and location assignment

Participants wore a GlobalSat DG-100 GPS tracker (GlobalSat, New Taipei City, Taiwan), with latitude and longitude collected every 30 s when a signal was attainable. The DG-100 tracker is considered an accurate device for measuring daily location patterns and has good spatial accuracy; the metrics of the DG-100 GPS metrics have been reported in other epidemiological studies [33]. Each participant’s home and school addresses were geocoded and incorporated into ArcGIS (ESRI, Inc., Redlands, CA) to create a 50-m circular buffer around the point resulting from geocoded the home address and a 15-m buffer around the geocoded school parcel. These buffer sizes were selected to minimize misclassification while considering potential errors caused be satellite interference, and are generally consistent with previous physical activity GPS studies [13, 34, 35]. Spatial analyses (methods published previously in [13]), were performed in PostgreSQL (PostgreSQL Global Development Group, Berkeley, CA) to classify each GPS point by location: at home (within the home or surrounding buffer), at school (within the school parcel or surrounding buffer), or all “other” locations (i.e., any location other than home and school). In this case, transport/trips would be classified in the “other” location.

#### Physical activity and sedentary time

Hip-worn ActiGraph accelerometers were used (models: 7164, 87.8%; GT1M, 8.0%; GT3X, 3.4%). Non-wear periods were defined as 30+ minute bouts of consecutive epochs with 0 accelerometer counts and subsequently excluded from analyses. GPS and accelerometer data were integrated to 30-s epochs and merged based on timestamps using a nearest neighbor approach (within up to a 30 s difference), and epochs with periods of missing GPS data or accelerometer non-wear time were removed from the dataset.

The Evenson cut points [36], which have excellent classification accuracy for measuring physical activity and sedentary time [37], were applied to physical activity counts within each 30-s epoch to classify MVPA and to 60-s epochs to classify sedentary time based on vertical axis accelerometer counts. The shorter epoch period for classifying MVPA has been shown to have greater validity than using 60-s epochs in youth [38]. The combined GPS and accelerometer data were then processed to create location-specific physical activity time, sedentary time, and sedentary bout patterns. For the bout pattern scoring, each epoch was first defined as occurring at home, at school, or at “other” locations. Sedentary bouts were defined as periods of sedentary time lasting ≥1 min. A sedentary bout ended when the epoch had an accelerometer count > 100 (no tolerance) or when the location (home, school, or other) changed for ≥2 consecutive GPS epochs. Next, location-specific sedentary bout pattern variables were scored, including: 1) total sedentary time; 2) prolonged time in sedentary bouts lasting ≥30 min; 3) mean bout duration, which represents the mean duration of all sedentary bouts; 4) period, the average duration between the end of a sedentary bout and the start of another within the wear time period spent at a location (calculated using the same methods described above for sedentary bouts); and 5) alpha, which represents an individual’s distribution/slope of sedentary bout lengths based on a power law function [39, 40]. Alpha is unit-less, with lower values reflecting more time in prolonged (longer) sedentary bout lengths [39, 40].

For the school location, variables were derived for school days only (e.g., average minutes/day of MVPA across school days). For the home and “other” locations, variables were derived for a “weighted week”, calculated as ([mean daily values across school days*5] + [mean daily values across non-school days*2]) ÷ 7, similar to previous protocols [13]. This was done to provide a better representation of a full week when the number of actual wear days on weekdays and weekends was imbalanced. Participants were required to have spent an average of ≥30 min/day in a location category (i.e., home, school, or other) as measured by the combined accelerometer wear time and GPS location data, to calculate each location-specific physical activity and sedentary time variable. Location-specific variables were recoded as missing for participants who did not spend ≥30 min/day in that location on average across days. These location wear time inclusion criteria were stricter than what were used in our previous analyses of location-based MVPA [13]. The stricter inclusion criteria were selected to better capture a representation of typical behavior in a location and eliminate momentary changes in behavior as adolescents transitioned across locations. It specifically aimed to improve estimates of the percent of time in the home location that was spent sedentary but was also likely to improve estimates of MVPA.

#### Dietary recall

Trained interviewers attempted three 24-h dietary recalls (two weekdays and one weekend day) via telephone for each participant on unannounced, random, non-consecutive days during the school year. Dietary data were collected and analyzed using the Nutrient Data System for Research software (version 2010) developed by the University of Minnesota Nutrition Coordinating Center [41]. The computer-assisted, interviewer-administered recalls were facilitated by an automated, four-stage, multi-pass technique [42, 43], which has acceptable validity for assessing dietary intake in youth as young as 8 years old [44] and has been implemented in national US dietary surveys [45]. Both the interviewers and participants had access to posters that displayed two-dimensional illustrations of cups, spoons, bowls, and other common food shapes to assist with portion size estimation.

The dietary behaviors of interest included daily energy intake (kcal), as well as other daily diet quality indicators such as added sugar (g), sodium (mg), fruits and vegetable (servings), high calorie beverages (number), sweet and savory snacks (number), whole grains (servings), fiber (g), fat calories (kcal), and saturated fat calories (kcal) [30]. Percent of fat and saturated fat were also calculated (fat kcal ÷ total kcal from models not per 100 kcal of energy intake*100). During recalls, participants were asked to report the location in which they consumed food/drinks during each eating episode (regardless of where it was purchased or prepared), which was used to derive location-specific dietary behaviors for at home, at school, and at “other” locations. For example, school-based dietary behaviors were defined as any food consumed at school regardless of where it was bought or made. The “other” location comprised all other locations, for example at work, after school programs, deli/take-out/store, restaurant/cafeteria/fast food, friend’s home, and party/reception/sporting event locations. For the school location, variables were derived for school days only, whereas for the home and “other” locations, variables were derived for a weighted week using the same equations that were used to calculate the weighted physical activity variables.

### Data analysis

All models were mixed-effects linear regression models, fitted with the “MIXED” command in SPSS version 24 (IBM SPSS Statistics, IBM Corporation), and included a random census block group intercept to account for the nesting of participants within census block groups. All models were adjusted for neighborhood walkability (low vs high), census-based median household income (low vs high), and adolescent and household characteristics including age, sex, race/ethnicity, and highest parental education level. All physical activity and sedentary models were adjusted for ActiGraph model, number of school and weekend days the participant wore the accelerometer, and average minutes/day of wear time. Models investigating dietary behaviors were additionally adjusted for participant height and weight and number of days of dietary recall.

To determine the magnitude of differences in physical activity and dietary behaviors across locations (i.e., home, school, “other”; Aim 1), location was entered as a categorical repeated-effects independent variable, and separate models were investigated for each behavior dependent variable using the aforementioned covariates. A second set of physical activity and sedentary time models evaluated differences in the proportion of time in each location that was spent in MVPA, total sedentary time, and time in 30+ minute sedentary bouts standardized per 60 min of wear time. Such proportional variables were not calculated for mean bout duration, period, and alpha since these variables are less likely to be affected by total time spent in a location. A second set of dietary behavior models evaluated the proportional differences in dietary behaviors as a function of energy intake in each location using nutrient densities (i.e., dietary value per 100 kcal of energy intake in each location). All Aim 1 models produced adjusted mean estimates of obesogenic behaviors in each location, as well as standard errors for these estimates. Post-hoc multiple comparisons were computed to determine if there were significant differences in these estimates and the 95% confidence intervals were reported. A more conservative *p*-value of *p* < .01 was used to provide evidence for significant differences between locations due to the large number of tests.

Associations of each behavior between each pair of locations were tested to investigate the extent to which a participant with more healthy behaviors in one location engaged in more healthy behaviors in other locations, relative to other participants (Aim 2). Each behavioral variable at “other” locations (e.g., “other” MVPA) was regressed on the same behavioral variable at home (e.g., home MVPA) and at school (e.g., school MVPA), and each behavioral variable at home was regressed on the same behavioral variable at school in mixed models. Both the independent and dependent behavioral variables were standardized to have a mean of zero and standard deviation of one to derive standardized regression coefficients. The magnitude of the standardized associations of behaviors between locations were interpreted as small (< .3) medium (.3–.5), and large (>.5) [46].

## Results

### Sample characteristics and descriptive statistics

The mean age was 14.1 (SD = 1.5) years for participants in the physical activity analysis and 14.1 (SD = 1.4) years for those with dietary behavior data*.* There was a total of 234 and 364 school locations in the physical activity and dietary analyses respectively. On average, participants wore the accelerometer for 5.19 (SD =1.30) days and 96.4% of participants had ≥3 wear days [47]. Sex, parental marital status, parental education, neighborhood walkability, family income, and site of recruitment were comparable between these overlapping groups and between these subgroups and the full TEAN sample (*N* = 928). However, a higher proportion of White non-Hispanic adolescents were included in the accelerometer/GPS sample than the dietary behavior sample (F [1780] = 5.76, *P* = 0.017) and the full TEAN sample (F [1919] = 7.68, *P* = .006). See Table 1 for a more detailed description of demographic characteristics for each of the samples. Participants had more device wear time at school (52.4%) compared to home (22.7%) and “other” locations (24.9%; An additional doc file shows this in more detail [Table S2 in Additional file 1]). Almost all participants in the dietary analyses (99.4%) completed all three dietary recalls.

**Table 1:** Participant and study characteristics

	Physical activity sample		Diet sample	
	*N*	Mean (SD) or %	*N*	Mean (SD) or %
Age	472	14.12 (1.47)	789	14.07 (1.41)
Sex				
Male	233	49.4	393	49.8
Female	239	50.6	396	50.2
Race/Ethnicity				
Non-white or Hispanic	135	28.7	271	34.7
White, non-Hispanic	335	71.3	511	65.3
Parent’s Marital Status				
Married or living with partner	391	83.0	656	83.5
Not married or living with partner	80	17.0	130	16.5
Parental Education				
Completion of college degree or higher	351	74.7	595	75.4
Other	119	25.3	191	24.2
Neighborhood Walkability				
Low walkability	257	54.4	401	50.8
High walkability	215	45.6	388	49.2
Approximate Annual Household Income				
< $50,000	67	14.7	101	13.4
$50,000 - < $100,000	176	38.7	312	41.4
≥ $100,000	212	46.6	341	45.2
Site				
Baltimore, MD-Washington, DC	228	48.3	425	53.9
Seattle-King County, WA	244	51.7	364	46.1

### Aim 1: differences in adolescent physical activity and dietary behavior across locations

About 50.4% of participants’ MVPA occurred at school (22.8 min), 35.1% occurred at “other” locations (15.9 min), and 14.5% occurred at home (6.6 min). However, in terms of proportion of time spent in MVPA (i.e., minutes of MVPA per 60 min of wear time), participants were most active at “other” locations (5.9 min/hour of wear time) compared to home (2.5 min/hour of wear time) and school locations (2.9 min/hour of wear time) (An additional doc file shows this in more detail [see Table S2 in Additional file 1]). Participants were most sedentary (including proportional to time in location) at school and proportionally least active at home compared to school and “other” locations. Additionally, time in 30+ minute sedentary bouts (including proportional to time in location) and mean bout duration were the highest, and alpha was the lowest, when participants were at school as compared to home and “other” locations. The period between sedentary bouts was lowest at home compared to school and “other” locations.

Regarding dietary behaviors, 61.1% of participants’ daily energy intake occurred at home (1293.0 kcal) and 25.2% at school (533.0 kcal), with the remaining 13.7% occurring at “other” locations (289.5 kcal) (An additional doc file shows this in more detail [see Table S3 in Additional file 2]). At “other” locations, restaurants/fast food/delis comprised 37.7% of the meals, friends’ houses comprised 24.3%, and sources such as other people’s homes and events comprised the remainder. Daily grams of added sugar intake and daily grams of fiber were proportionately the least favorable (i.e., nutritionally poorer per 100 kcal of energy intake) at “other” locations compared to home and school locations. Daily servings of fruit and vegetables and daily servings of whole grains were proportionately less nutritionally favorable at “other” locations compared to at school, but not at home. Daily number of high calorie beverages was proportionately greater at “other” locations compared to at home, but not at school. Daily calories from fat and saturated fat were proportionally greater at “other” locations compared to at home and at school. For example, the percent of calories consumed from fat was 32.0, 28.9 and 32.4% for the school, home and “other” locations respectively. The daily number of sweet and savory snacks consumed was proportionately the highest at school compared to at home, but not at “other” locations. Sodium intake was proportionally comparable across locations.

### Aim 2: school-home, school-other, and home-other associations of adolescent physical activity and dietary behavior

The magnitude of associations of each physical activity and dietary behavior variable between pairs of locations, as indicated by standardized regression coefficients, was small (βs < .3) for 93% of the associations and moderate (βs = .3–.5) for 7% of the associations (Table 2). Physical activity, sedentary time, prolonged sedentary time, mean bout duration, and period were more strongly associated between locations, while alpha was generally less strongly associated between locations. The magnitudes of associations among physical activity and sedentary variables between locations were similar for each pair of locations, but differences were observed within the dietary behaviors. For example, dietary behaviors were more strongly associated between the school and home locations than between the school and “other”, and home and “other” locations. Sodium, fruits and vegetable servings, number of high calorie beverages, whole grains, fiber, diet quality, and fat calories were more strongly associated between locations than added sugar intake, sweet and savory snacks, and fat calories consumed.

**Table 2:** Associations of adolescent physical activity and dietary behavior between locations

	β [95% CI]		
	School^a^-home^b^	School^a^-other^b^	Home^b^-other^b^
**Physical activity variables**			
MVPA, minutes/day	0.17* [0.10, 0.27]	0.16* [0.08, 0.25]	0.06 [−0.03, 0.16]
Sedentary time, minutes/day	0.11* [0.06, 0.15]	0.16* [0.11, 0.22]	0.21* [0.09, .034]
Time in 30+ min sedentary bouts, minutes/day	0.10 [0.01, 0.20]	0.13* [0.03, 0.23]	0.10 [< 0.001, 0.20]
Mean bout duration, minutes/day	0.06 [−0.003, 0.12]	0.20* [0.12, 0.28]	0.17* [0.05, 0.30]
Period, minutes/day	0.30* [0.18, 0.34]	0.13 [0.03, 0.22]	0.26* [0.15, 0.36]
Alpha	0.02 [−0.07, 0.12]	0.15* [0.06,0 .23]	0.08 [−0.02, 0.18]
**Dietary variables**			
Added sugar, grams	0.17* [0.04, 0.29]	0.05 [−0.01, 0.11]	0.03 [−0.003, 0.06]
Sodium, mg	0.18* [0.06, 0.30]	0.04 [−0.02, 0.09]	0.05* [0.02, 0.08]
Fruits and vegetables, servings	0.31* [0.12, 0.42]	0.02 [−0.05, 0.08]	0.02 [−0.02, 0.06]
High calorie beverages, number	0.22* [0.12, 0.34]	−0.01 [− 0.06, 0.04]	0.08* [0.04, 0.11]
Sweet and savory snacks, number	0.10 [0.01, 0.20]	0.03 [−0.01, 0.07]	0.03 [−0.004, 0.05]
Whole grains, servings	0.23* [0.12, 0.33]	0.07* [0.04, 0.09]	0.03 [−0.001, 0.03]
Fiber, grams	0.52* [0.38, 0.65]	0.09* [0.05, 0.13]	0.04* [0.03, 0.07]
Fat calories, kcal	0.11 [−0.01, 0.23]	0.04 [−0.02, 0.09]	0.04 [0.001, 0.08]
Saturated fat calories, kcal	0.23* [0.10, 0.36]	−0.01 [− 0.07, 0.04]	0.04 [0.003, 0.07]

β = standardized regression coefficients with both the independent and dependent variables standardized to have a mean of zero and standard deviation of 1

Second variable in column (Variable1-Variable2) represents the dependent variable

All models adjusted for participant age, sex, race/ethnicity, parent education, and study design factors. Physical activity variables were additionally adjusted for time spent at each location, accelerometer model, number of days of accelerometer wear, number of school days, and accelerometer wear time. Dietary variables were additionally adjusted for participant height, weight, number of days of dietary recall, and energy intake at each location

^a^On school days only

^b^Calculated for a weighted week (weekdays*5 + weekend days*2)/7

*p < .01

## Discussion

The current study investigated the relative contribution of home, school, and “other” locations to physical activity, sedentary time, and dietary behaviors in adolescents. The main findings were that adolescents’ physical activity and dietary behaviors differed based on the location/environment they were in. These findings suggest that health behaviors of adolescents vary by the environments in which they spend time, supporting a hypothesis of partial environmental determination of behavior in addition to intrapersonal determination [10]. The home and school locations appeared to support lower amounts of physical activity and greater total sedentary time, but healthier diets in adolescents, on average, as compared to “other” locations. The school location specifically appeared to support greater prolonged bouts of sedentary time and mean bout durations, as well as potentially less-healthy snacking. The mostly small associations of health behaviors between locations provided additional support for the notion that location-based factors play a major role in adolescents’ health behaviors. If individual factors were strong, such as preferences and motivation, substantial correlations of behaviors across locations would be expected. The overall pattern of results reinforces the notion that health behavior interventions should consider that healthy eating and active living opportunities differ by setting type. One implication is that distinct intervention strategies are likely needed in each location and combining strategies to support health behaviors in multiple locations is likely to be most effective.

Youth are recommended to obtain ≥60 min of daily MVPA, and schools are recommended to provide ≥30 min of MVPA during school [48, 49]. This study found that on average, adolescents obtained approximately 20 min of MVPA at school, which is only one-third of the recommended amounts of daily MVPA and two-thirds of the recommended amounts of school MVPA. This finding was also shown in a previous study in this sample focusing on MVPA [13]. However, the more refined methods used in this current paper (i.e., requiring a minimum amount of time to be spent in a location to derive MVPA in that location) showed that adolescents were proportionally least active at home rather than at school. Thus, though adolescents spend large amounts of time at home and school, they appear to engage in very low levels of MVPA in both of these locations, particularly when compared to “other” locations. These results are in agreement with similar studies and suggest that efforts are needed to increase adolescents’ physical activity both during and outside of school [11, 13–17]. Numerous school-based approaches exist for supporting physical activity at school, including quality physical education, before- and after-school physical activities, and classroom-based physical activity [50].

The present study was among the first to investigate sedentary time and patterns across locations. Present findings indicated that schools not only support a high amount of total sedentary time, approximately 40 min per hour, they also support adolescents to be sedentary for long periods without interruption (i.e., more prolonged bouts). Though evidence about the relation of prolonged sedentary bouts to health outcomes in youth is equivocal [51–53], there are some studies linking prolonged sedentary patterns with higher weight status and cardiometabolic risk [21]. School teaching models for which longer sedentary durations are thought to facilitate learning may inadvertently contribute to obesogenic outcomes. In contrast, multiple studies have documented the benefits of light activity and MVPA on learning and classroom behavior [54, 55]. Taken together, these findings suggest that school-based strategies to reduce prolonged sedentary time are warranted. Such strategies are likely to involve modifications to school environments and policies. Active classroom programs [56] and active school design guidelines [57] exist for promoting physical activity and reducing sedentary time at school, such as organizing classroom furniture to allow for greater movement. Additional research supports larger classroom sizes, standing desks, and other ergonomically-friendly furniture to support movement [57]. Teachers may also need to adapt their teaching practices to support frequent interruptions from sedentary time. With regards to home sedentary time, screen time is likely a large and increasing driver [58] and multilevel intervention strategies are likely needed that reduce screen time as well as incorporate standing and movement into screen-based activities [59].

Generally, more-favorable dietary behaviors were most evident at home and school, while poorer (more obesogenic) dietary behaviors were most evident in “other” locations. Adolescents may have greater access to healthier foods and less autonomy when at home and school as compared to “other” locations, partly due to greater parental involvement in food availability and feeding at home as well as improvements in national nutrition standards for healthier lunches at school [30, 60]. Limited availability of healthy food options at “other” locations in conjunction with adolescents having greater autonomy over their food choices in these locations may confer risk for obesogenic dietary behaviors at “other” locations. Access to vending machines or unhealthy à la carte food options may have contributed to higher rates of unhealthy snacking in school locations [25, 61]. Policies and practices that target the elimination, replacement, or reduction of these unhealthy food options at school might mitigate these behaviors in this location. Given that the “other” location included any setting outside the home and school (e.g., restaurants, fast food places, and friends’ houses), various specific locations likely impacted dietary behaviors differently, but these differences were not investigated in the current study. However, most meals in “other” locations occurred at restaurants/fast foods/delis, which can often provide greater unhealthy and limited healthy food options as compared to home [26–28]. It may prove beneficial to support adolescents to improve their skills for engaging in healthy eating behaviors when they are away from home and school (e.g., bringing healthy snacks with them, choosing nutritious food options at restaurants).

The mostly small associations of health behaviors between each pair of locations suggests little carryover of health behaviors from one location to another. In other words, adolescents who were more healthful (than their peers) in one location were not necessarily more healthful in other locations, and knowing someone’s health behaviors in one location did not allow strong predictions of their behaviors in another location. The potential exception to this was higher associations between the home and school locations for some of the dietary behaviors. This could reflect adolescents bringing food from home to consume at school. Nonetheless, an individual’s propensity towards a health-promoting or health-compromising behavior may be better understood by recognizing the context of the location in which it occurs than by only considering characteristics of the individual. This also suggests that environmental factors may undermine health promotion efforts in some locations. Such factors in relation to diet include health-compromising marketing or messages (e.g., fast food advertisements) [62] and in relation to physical activity include a lack of opportunities (e.g., physical education) or resources (e.g., sidewalks), all of which have important policy implications.

### Limitations and future directions

This study was strengthened by its use of device-measured data via accelerometry and GPS to quantify the magnitude of differences in physical activity and sedentary time across locations. Requiring only 2 days of accelerometer data may not have captured a representation of all participant's activity. However, the inclusion of ≥1 weekday and ≥ 1 weekend day was likely to improve the activity estimates, and most (96.4%) participants had ≥3 days of data which has been shown to provide reliable estimates of habitual activity [47]. While criteria were set forth to address GPS satellite interference (e.g., large buffers, up to 2 epochs allowed outside of the location before breaking up a bout), it is possible that errant GPS scatter may have incorrectly recorded a participant’s physical activity in the wrong location. Future studies could consider additional strategies to test the impact of and to minimize GPS error. All dietary variables were based on participant recall, potentially limiting the quality of these data, though the dietary measures used were among the most valid of available options [42–44]. Another limitation was that location-specific dietary behaviors were categorized by the location in which meals were *consumed*, so it was not possible to compare school lunches to home-prepared lunches eaten at school. Since the “other” location was broadly defined, limited inferences can be made about the specific sources (e.g., transport/trips, restaurants, gyms, parks, friend’s houses, work centers, etc.) influencing health behaviors in “other” locations. Transport between locations was included in the “other” location so time in active transportation may have contributed to greater MVPA in this location. This inclusion limits conclusions regarding the effect of “other” locations on physical activity/sedentary time since transportation is not a location. Although physical activity was higher and dietary behaviors poorer in “other” locations, the specific “other” locations that supported more physical activity/less sedentary time and less healthy diets were likely different (e.g., recreation areas vs. food outlets). This study was limited by a lack of information on the details of the locations within the “other” category. However, a previous study showed that a large portion of the “other” category comprises the home and school neighborhood (including active transport [63]), particularly in regard to MVPA (53% of “other” location MVPA occurred in the home or school neighborhood) [13]. Still, future research should aim to parse out the specific locations within the “other” category that support physical activity and healthy dietary behaviors to better inform location-tailored interventions.

Present findings do not suggest these locations cause healthy versus obesogenic behaviors. Rather, present findings highlight critical locations in which adolescents more often exhibit obesogenic behaviors via associative and comparative analyses. It’s also important to recognize the potential of selective daily mobility, which refers to the notion that individuals seek out certain locations for various reasons [64]. In the present study, mobility bias may be particularly relevant to the “other” location. For example, adolescents who are more active may deliberately seek out parks to exercise, and thus the location may be supportive of physical activity because it fulfills the desire the exercise rather than actually influencing the participant to exercise. However, in this example, the location (park) still appears to be important to supporting health because it is unknown if the exercise would have occurred without the park. Although the present study aimed to investigate differences in behaviors across locations, there was substantial variation within location (across participants) which could be explained by individual psychosocial differences as well as likely variation in environmental features within these locations (e.g., schools with different healthy and unhealthy food access) and/or context-specific psychosocial factors, warranting future research. For instance, an adolescent’s motivation, self-efficacy, and social support to engage in healthy or unhealthy behaviors may differ across locations and in response to environmental features within a location. With further setting-specific research, future interventions can be tailored to the specific environment as well as individualized based on adolescent psychosocial factors.

## Conclusions

Adolescent physical activity and dietary behaviors differed across home, school, and “other” locations. School settings may contribute to adolescents being more sedentary than they might be otherwise. “Other” locations were related to more physical activity but less-healthful dietary behaviors. Present findings indicate that adolescents’ health behaviors should be considered in the context of their location. Health promotion programs and health behavior interventions should therefore include location-specific strategies to maximize healthy decision-making within each of the many locations where adolescents spend large amounts of time. Future studies should examine the interplay between individual-level factors, environmental factors, and specific locations in impacting obesogenic health behaviors to further refine location-targeted health behavior interventions.

## Supplementary information

**Additional file 1: Table S2.** Differences in physical activity behaviors across school, home, and other locations.

**Additional file 2: Table S3.** Differences in dietary behaviors across school, home, and other locations.

**Additional file 3.** Title of data: STROBE-nut Statement—checklist of items that should be included in reports of observational studies. Description of data: Includes STROBE-nut Checklist for the study.

## Data Availability

The data and research materials can be obtained by contacting the third and last author of this paper.

## Abbreviations

US: United States
GPS: Global positioning system
MVPA: Moderate-to-vigorous physical activity
PALMS: Personal activity and location measurement system
TEAN: Teen environment and neighborhood
