# Supplementary material for: Differences in adolescent activity and dietary behaviors across home, school, and other locations warrant location-specific intervention approaches
Source: Int J Behav Nutr Phys Act. 2020 Sep 29;17:123. doi: 10.1186/s12966-020-01027-1 (PMC7526379; doi:10.1186/s12966-020-01027-1)
Supplement: Supplementary file 1 — Additional file 1: Table S2. Differences in physical activity behaviors across school, home, and other locations. [file 12966_2020_1027_MOESM1_ESM.docx]

| Table 2.  *Differences in physical activity behaviors across school, home, and other locations* | | | | | | |
| --- | --- | --- | --- | --- | --- | --- |
|  | Daily observed mean (SE) in each location | | | A vs B Pairwise Differences [95%CI], *p*-value | A vs C Pairwise Differences  [95%CI], *p*-value | B vs C Pairwise Differences  [95%CI], *p*-value |
|  | A. School^a^ | B. Home^b^ | C. Other^b^ |  |  |  |
| Wear Time, minutes | 445.8 (23.3) | 192.6 (23.7) | 211.3 (23.7) | 253.2 [236.6, 270.7], *p* < .001 | 234.5 [217.3, 251.6], *p* < .001 | -18.67 [-38.0, .83], *p* = .06 |
| **Physical activity variables** |  |  |  |  |  |  |
| MVPA, minutes | 22.77 (1.83) | 6.56 (1.73) | 15.89 (1.85) | 16.21 [14.75, 17.68], *p* < .001 | 6.88 [4.94, 8.82], *p* < .001 | -9.33 [-10.88, -7.78], *p =* .98 |
| Sedentary time, minutes | 307.2 (15.7) | 136.8 (15.8) | 136.7 (15.8) | 170.4 [158.8, 182.0], *p* < .001 | 170.6 [159.2, 181.9], *p* <.001 | 0.15 [-12.13, 12.43], *p* < .001 |
| Time in 30+ min sedentary bouts, minutes | 78.87 (5.97) | 26.71 (5.47) | 30.12 (5.58) | 52.16 [46.54, 57.78], *p* < .001 | 48.76 [42.74, 54.79], *p* <.001 | -3.40 [-7.15, 0.36], *p* = .08 |
| Mean bout duration, minutes | 5.34 (0.34) | 4.26 (0.35) | 4.34 (0.35) | 1.08 [0.81, 1.35], *p* < .001 | 1.00 [0.76, 1.24], *p* < .001 | -0.08 [-0.36, 0.20], *p =* .56 |
| Period, minutes | 2.74 (0.16) | 2.33 (0.16) | 2.94 (0.17) | 0.41 [0.31, 0.50], *p* < .001 | -0.21 [-0.35, -0.06], *p* = .01 | -0.61 [-0.76, -0.46], *p* < .001 |
| Alpha | 1.84 (0.05) | 2.23 (0.07) | 2.17 (0.05) | -0.39 [-0.50, -0.28], *p* < .001 | -0.33 [-0.37, -0.29], *p* < .001 | 0.06 [-0.05, 0.17], *p* = .27 |
| **Proportional physical activity variables per 60 minutes of wear time** |  |  |  |  |  |  |
| MVPA, minutes | 2.87 (.38) | 2.46 (.39) | 5.88 (.45) | 0.41 [0.16, 0.66], *p =* .001 | -3.01 [-3.54, -2.49], *p* < .001 | -3.42 [-3.97, -2.87], *p* < .001 |
| Sedentary time, minutes | 40.69 (1.14) | 38.55 (1.15) | 34.93 (1.16) | 2.13 [1.39, 2.88], *p <* .001 | 5.76 [4.96, 6.56], *p < .*001 | 3.63 [2.79, 4.46], *p < .*001 |
| Time in 30+ min sedentary bouts, minutes | 10.04 (1.09) | 5.78 (1.07) | 5.83 (1.07) | 4.26 [3.46, 5.06], *p* < .001 | 4.20 [3.41, 5.00], *p* < .001 | -0.06 [-0.78, .07], *p* = .88 |
| All models adjusted for participant age, sex, race/ethnicity, parent education, and study design factors, accelerometer model, number of days of accelerometer wear, and number of school days.  ^a^On school days only  ^b^Calculated for a weighted week (weekdays*5+weekend days*2)/7 | | | | | | |
