# Supplementary material for: Differences in adolescent activity and dietary behaviors across home, school, and other locations warrant location-specific intervention approaches
Source: Int J Behav Nutr Phys Act. 2020 Sep 29;17:123. doi: 10.1186/s12966-020-01027-1 (PMC7526379; doi:10.1186/s12966-020-01027-1)
Supplement: Supplementary file 2 — Additional file 2: Table S3. Differences in dietary behaviors across school, home, and other locations. [file 12966_2020_1027_MOESM2_ESM.docx]

| Table 3.  *Differences in dietary behaviors across school, home, and other locations* | | | | | | |
| --- | --- | --- | --- | --- | --- | --- |
|  | Daily observed mean (SE) in each location | | | A vs B Pairwise Differences  [95%CI], *p*-value | A vs C Pairwise Differences  [95%CI], *p*-value | B vs C Pairwise Differences  [95%CI], *p*-value |
|  | A. School^a^ | B. Home^b^ | C. Other^c^ |  |  |  |
| **Dietary behaviors** |  |  |  |  |  |  |
| Energy intake, kcal | 533.0 (12.5) | 1293.0 (19.5) | 289.5 (12.5) | -761.0 [-806.3, -715.6], *p* < .001 | 243.6 [209.0, 278.2], *p* < .001 | 1004.5 [959.2, 1049.3], *p* < .001 |
| Added sugar, grams | 19.59 (.73) | 45.44 (1.17) | 13.16 (.71) | -25.85 [-28.50, -23.21], *p* < .001 | 6.42 [4.50, 8.34], *p* < .001 | 32.27 [29.66, 34.49], *p* < .001 |
| Sodium, mg | 914.0 (24.3) | 2139.0 (36.6) | 482.7 (23.0) | -1225.0 [-1311.3, -1138.7], *p* < .001 | 431.3 [365.6, 497.0], *p* < .001 | 1656.3 [1571.4, 1741.2] *p* < .001 |
| Fruits and vegetables, servings | 0.98 (0.04) | 2.38 (.07) | 0.42 (.04) | -1.41 [-1.56, -1.26], *p* < .001 | 0.56 [0.45, 0.66], *p* < .001 | 1.97 [1.82, 2.11], *p* < .001 |
| High calorie beverages, number | 0.30 (.02) | 0.61 (.04) | 0.24 (.02) | -0.31 [-0.39, -0.26], *p* < .001 | 0.07 [0.01, 0.12], *p* = .03 | 0.37 [0.29,0 .45], *p* < .001 |
| Sweet and savory snacks, number | 0.85 (.04) | 1.36 (.05) | 0.34 (.02) | -0.51 [-0.64, -0.39], *p* < .001 | 0.52 [0.42, 0.61], *p* < .001 | 1.03 [0.92, 1.13], *p* < .001 |
| Whole grains, servings | 0.49 (.03) | 1.14 (.05) | 0.10 (.01) | -0.65 [-0.75, -0.54], *p* < .001 | 0.39 [0.33, 0.45], *p* < .001 | 1.04 [0.05, 1.13], *p* < .001 |
| Fiber, grams | 4.42 (.12) | 10.21 (.21) | 1.81 (.09) | -5.79 [-6.27, -5.31], *p* < .001 | 2.62 [2.32, 2.91], *p* < .001 | 8.41 [7.96, 8.86], *p* < .001 |
| Fat calories, kcal | 170.3 (4.8) | 374.1 (7.0) | 93.6 (5.0) | -203.9 [-220.5, -187.2], *p* < .001 | 76.68 [63.25, 90.10], *p* < .001 | 280.5 [263.7, 297.4], *p* < .001 |
| Saturated fat calories, kcal | 56.59 (1.74) | 139.9 (2.9) | 33.27 (1.84) | -83.27 [-89.94, -76.60], *p* < .001 | 23.32 [18.36, 28.29], *p* < .001 | 106.6 [99.82, 111.4], *p* < .001 |
| **Proportional dietary behaviors per 100 kcal of energy intake** |  |  |  |  |  |  |
| Added sugar, grams | 3.71 (.12) | 3.48 (.08) | 4.85 (.18) | 0.23 [-0.03, 0.49], *p* = .08 | -1.14 [-1.56, -0.72], *p* < .001 | -1.37 [-1.74, -1.0], *p* < .001 |
| Sodium, mg | 171.1 (2.8) | 167.4 (1.9) | 163.6 (3.8) | 3.64 [-2.55, 9.83], *p* = .25 | 7.43 [-1.60, 16.45], *p* = .11 | 3.79 [-4.27, 11.85], *p* = .36 |
| Fruits and vegetables, servings | 0.20 (.01) | 0.19 (.01) | 0.16 (.01) | 0.00 [-0.02, 0.02], *p* = .69 | 0.04 [0.01, 0.07], *p* = .004 | 0.04 [0.01, 0.06], *p* = .003 |
| High calorie beverages, number | 0.07 (.01) | 0.05 (.00) | 0.09 (.01) | 0.02 [0.01, 0.03], *p* = .01 | -0.02 [-0.04, 0.00], *p* = .03 | -0.04 [-0.06, -0.02], *p* < .001 |
| Sweet and savory snacks, number | 0.17 (.01) | 0.11 (.00) | 0.14 (.01) | 0.07 [0.04, 0.09], *p* < .001 | 0.03 [0.00, 0.06], *p* = .05 | -0.04 [-0.06, -0.02], *p* < .001 |
| Whole grains, servings | 0.10 (.01) | 0.09 (.00) | 0.04 (.01) | 0.01 [0.00, 0.03], *p* = .08 | 0.06 [0.04, 0.07], *p* < .001 | 0.05 [0.04, 0.06], *p* < .001 |
| Fiber, grams | 0.88 (.02) | 0.80 (.01) | 0.65 (.02) | 0.08 [0.03, 0.12], *p* = .001 | 0.23 [0.17, 0.28], *p* < .001 | 0.15 [0.11, 0.20], *p* < .001 |
| Fat calories, kcal | 31.19 (.40) | 28.31 (.28) | 33.32 (.62) | 2.88 [1.96, 3.80], *p* < .001 | -2.14 [-3.55, -0.72], *p* = .003 | -5.02 [-6.32, -3.71], *p* < .001 |
| Saturated fat calories, kcal | 10.26 (.19) | 10.50 (.14) | 11.67 (.27) | -0.24 [-0.68, 0.19], *p* = .27 | -1.41 [-2.05, -.78], *p* < .001 | -1.17 [-1.75, -.59], *p* <.001 |
| All models adjusted for participant age, sex, race/ethnicity, parent education, and study design factors, participant height and weight, and number of days of dietary recall.  ^a^On school days only  ^b^Calculated for a weighted week (weekdays*5+weekend days*2)/7 | | | | | | |
