# Supplementary material for: Differences in adolescent activity and dietary behaviors across home, school, and other locations warrant location-specific intervention approaches
Source: Int J Behav Nutr Phys Act. 2020 Sep 29;17:123. doi: 10.1186/s12966-020-01027-1 (PMC7526379; doi:10.1186/s12966-020-01027-1)
Supplement: Supplementary file 3 — Additional file 3. Title of data: STROBE-nut Statement—checklist of items that should be included in reports of observational studies. Description of data: Includes STROBE-nut Checklist for the study. [file 12966_2020_1027_MOESM3_ESM.docx]

STROBE-nut Statement—checklist of items that should be included in reports of observational studies

|  | Item No. | Recommendation | Page  No. | Relevant text from manuscript |
| --- | --- | --- | --- | --- |
| **Title and abstract** | 1 | 1. Indicate the study’s design with a commonly used term in the title or the abstract   (Nut-1: State the dietary/nutritional assessment methods used in the title, abstract, keywords) | 1-2 | Cross-sectional study; 3 day dietary recall |
|  |  | (*b*) Provide in the abstract an informative and balanced summary of what was done and what was found | 2 | Abstract |
| Introduction | | | |  |
| Background/rationale | 2 | Explain the scientific background and rationale for the investigation being reported | 4-6 | Examination of how locations relate to health behaviors is warranted given that opportunities for engaging in active living and health eating can differ substantially across locations and the relative influence of location versus individual factors on health behaviors is still unclear |
| Objectives | 3 | State specific objectives, including any prespecified hypotheses | 6 | This cross-sectional study aimed to examined whether physical activity, sedentary time, and indicators of healthy and unhealthy dietary behaviors differed across home, school, and other locations as captured objectively (physical activity and sedentary time) or from multiple 24-hour recalls with reported eating location information (dietary behaviors). |
| Methods | | | |  |
| Study design | 4 | Present key elements of study design early in the paper | 6 | Participants for the present analyses were part of the Teen Environment and Neighborhood (TEAN) Study examining the relation of built environmental factors with physical activity and dietary behaviors |
| Setting | 5 | Describe the setting, locations, and relevant dates, including periods of recruitment, exposure, follow-up, and data collection  (Nut-5: Describe any characteristics of the study settings that might affect the dietary intake or nutritional status of the participants, if applicable.) | 6-8 | Adolescents were recruited from the Seattle/King County, Washington and Maryland-Washington, DC metropolitan areas from 2009-2011 during months school was in session. |
| Participants | 6 | (*a*) *Cohort study*—Give the eligibility criteria, and the sources and methods of selection of participants. Describe methods of follow-up  *Case-control study*—Give the eligibility criteria, and the sources and methods of case ascertainment and control selection. Give the rationale for the choice of cases and controls  *Cross-sectional study*—Give the eligibility criteria, and the sources and methods of selection of participants | 7 | These participants were part of the Teen Environment and Neighborhood (TEAN) observational study of neighborhood environment and physical activity (Sallis et al., 2018). |
|  |  | (*b*) *Cohort study*—For matched studies, give matching criteria and number of exposed and unexposed  *Case-control study*—For matched studies, give matching criteria and the number of controls per case |  | N/A |
| Variables | 7 | Clearly define all outcomes, exposures, predictors, potential confounders, and effect modifiers. Give diagnostic criteria, if applicable  (Nut-7.1: Clearly define foods, food groups, nutrients, or other food components.)  (nut-7.2. When using dietary patterns or indices, describe the methods to obtain them and their nutritional properties.) | 8-12 | “Measures” sections; including details in “Dietary recall” section. |
| Data sources/ measurement | 8* | For each variable of interest, give sources of data and details of methods of assessment (measurement). Describe comparability of assessment methods if there is more than one group  nut-8.1. Describe the dietary assessment method(s), e.g., portion size estimation, number of days and items recorded, how it was developed and administered, and how quality was assured. Report if and how supplement intake was assessed.  nut-8.2. Describe and justify food composition data used. Explain the procedure to match food composition with consumption data. Describe the use of conversion factors, if applicable.  nut-8.3. Describe the nutrient requirements, recommendations, or dietary guidelines and the  evaluation approach used to compare intake with the dietary reference values, if applicable.  nut-8.4. When using nutritional biomarkers, additionally use the STROBE Extension for Molecular Epidemiology (STROBE-ME). Report the type of biomarkers used and their usefulness as dietary exposure markers.  nut-8.5. Describe the assessment of nondietary data (e.g., nutritional status and influencing factors) and timing of the assessment of these variables in relation to dietary assessment.  nut-8.6. Report on the validity of the dietary or nutritional assessment methods and any internal or external validation used in the study, if applicable. | 8-12 | Applicable information included in the “Measures” sections and “Dietary Recall” subsection |
| Bias | 9 | Describe any efforts to address potential sources of bias  (nut-9. Report how bias in dietary or nutritional assessment was addressed, e.g., misreporting, changes in habits as a result of being measured, or data imputation from other sources.) | 11 | Dietary recall methods: calls on random days; portion aides provided to assist with accurate recall reporting. |
| Study size | 10 | Explain how the study size was arrived at | 7-8 | See details for how we reached our sample size. |

Continued on next page

| Quantitative variables | 11 | Explain how quantitative variables were handled in the analyses. If applicable, describe which groupings were chosen and why  nut-11. Explain the categorization of dietary/nutritional data (e.g., use of N-tiles and handling of nonconsumers) and the choice of reference category, if applicable. | 8-12  11 | Measures sections described quantitative variables for MVPA, sedentary variables, #days in the study, and height and weight  We inform how dietary variables were calculated using the NDSR. |
| --- | --- | --- | --- | --- |
| Statistical methods | 12 | 1. Describe all statistical methods, including those used to control for confounding   nut-12.1. Describe any statistical method used to combine dietary or nutritional data, if applicable.  nut-12.2. Describe and justify the method for energy adjustments, intake modeling, and use of weighting factors, if applicable.  nut-12.3. Report any adjustments for measurement error, i.e., from a validity or calibration study. | 12-13 | See applicable information in the “Data Analysis” section |
|  |  | (*b*) Describe any methods used to examine subgroups and interactions | 13-14 | We compared subsamples to each other and larger TEAN sample |
|  |  | (*c*) Explain how missing data were addressed | 7-8, 10 | Those with missing data excluded |
|  |  | (*d*) *Cohort study*—If applicable, explain how loss to follow-up was addressed  *Case-control study*—If applicable, explain how matching of cases and controls was addressed  *Cross-sectional study*—If applicable, describe analytical methods taking account of sampling strategy | 12 | All models were adjusted for neighborhood walkability (low vs high), census-based median household income (low vs high), and adolescent and household characteristics including age, sex, race/ethnicity, and highest parental education level. |
|  |  | (*e*) Describe any sensitivity analyses |  | N/A |
| Results | | | | |
| Participants | 13* | 1. Report numbers of individuals at each stage of study—eg numbers potentially eligible, examined for eligibility, confirmed eligible, included in the study, completing follow-up, and analysed   nut-13. Report the number of individuals excluded based on missing, incomplete, or implausible dietary/nutritional data. | 7-8 | “Participants and procedures” section |
|  |  | (b) Give reasons for non-participation at each stage |  | N/A |
|  |  | (c) Consider use of a flow diagram |  | N/A |
| Descriptive data | 14* | 1. Give characteristics of study participants (eg demographic, clinical, social) and information on exposures and potential confounders   nut-14. Give the distribution of participant characteristics across the exposure variables if applicable. Specify if food consumption of total population or consumers only were used to obtain results. | 13-14 | Sample characteristics are presented in Table 1, on page 14.  N/A |
|  |  | (b) Indicate number of participants with missing data for each variable of interest | 7-8 | See “Participants and procedures” section |
|  |  | (c) *Cohort study*—Summarise follow-up time (eg, average and total amount) |  | N/A |
| Outcome data | 15* | *Cohort study*—Report numbers of outcome events or summary measures over time |  | N/A |
|  |  | *Case-control study—*Report numbers in each exposure category, or summary measures of exposure |  | N/A |
|  |  | *Cross-sectional study—*Report numbers of outcome events or summary measures | 15-17 | See “Aim 1” and “Aim 2” Results as well as Tables 2-3 in Additional Files. |
| Main results | 16 | 1. Give unadjusted estimates and, if applicable, confounder-adjusted estimates and their precision (eg, 95% confidence interval). Make clear which confounders were adjusted for and why they were included   nut-16. Specify if nutrient intakes are reported with or without inclusion of dietary supplement intake, if applicable. | 15-17 | See “Aim 1” and “Aim 2” Results as well as Tables 2-3 in Additional Files. Some variables adjusted for time and energy intake.  N/A |
|  |  | (*b*) Report category boundaries when continuous variables were categorized |  | N/A |
|  |  | (*c*) If relevant, consider translating estimates of relative risk into absolute risk for a meaningful time period |  | N/A |

Continued on next page

| Other analyses | 17 | Report other analyses done—eg analyses of subgroups and interactions, and sensitivity analyses  nut-17. Report any sensitivity analysis (e.g., exclusion of misreporters or outliers) and data imputation, if  applicable. |  | N/A |
| --- | --- | --- | --- | --- |
| Discussion | | | | |
| Key results | 18 | Summarise key results with reference to study objectives | 15-18 | See “Results” section and first paragraph of “Discussion” Section |
| Limitations | 19 | Discuss limitations of the study, taking into account sources of potential bias or imprecision. Discuss both direction and magnitude of any potential bias  nut-19. Describe the main limitations of the data sources and assessment methods used and implications for the interpretation of the findings. | 21-23 | See “Limitations” section. |
| Interpretation | 20 | Give a cautious overall interpretation of results considering objectives, limitations, multiplicity of analyses, results from similar studies, and other relevant evidence  nut-20. Report the nutritional relevance of the findings, given the complexity of diet or nutrition as an exposure. | 23 | See “Conclusion” section. |
| Generalisability | 21 | Discuss the generalisability (external validity) of the study results | 22 | See “Limitations” section |
| Other information | |  | | |
| Funding | 22 | Give the source of funding and the role of the funders for the present study and, if applicable, for the original study on which the present article is based | 3 | See “Funding” section in “Declarations” |

*Give information separately for cases and controls in case-control studies and, if applicable, for exposed and unexposed groups in cohort and cross-sectional studies.

**Note:** An Explanation and Elaboration article discusses each checklist item and gives methodological background and published examples of transparent reporting. The STROBE checklist is best used in conjunction with this article (freely available on the Web sites of PLoS Medicine at http://www.plosmedicine.org/, Annals of Internal Medicine at http://www.annals.org/, and Epidemiology at http://www.epidem.com/). Information on the STROBE Initiative is available at www.strobe-statement.org.
